# Prediction Factors of 6-Month Poor Prognosis in Acute Myocardial Infarction Patients

**DOI:** 10.3389/fcvm.2020.00130

**Published:** 2020-08-13

**Authors:** Jianhua Yao, Yuan Xie, Yang Liu, Yu Tang, Jiahong Xu

**Affiliations:** ^1^Department of Cardiology, Shanghai Tenth People's Hospital, Tongji University School of Medicine, Shanghai, China; ^2^Department of Cardiology, Tongji Hospital, Tongji University School of Medicine, Shanghai, China

**Keywords:** acute myocardial infarction, prognosis, death, readmission, biomarker

## Abstract

**Background:** Acute myocardial infarction (AMI) is among the leading causes of death worldwide. Patients with AMI may have the risk of developing recurrent cardiovascular events leading to rehospitalization or even death. The present study aimed to investigate the prediction factors of poor prognosis (mortality and/or readmission) after AMI during a 6-month follow-up.

**Methods:** A total of 206 consecutive patients hospitalized for the first visit with AMI were enrolled. Data collection included demographic characteristics, medical history, clinical information, laboratory results, and oral medications within 24 h of admission. At 1, 3, and 6 months after discharge, AMI patients were followed up to assess the occurrence of composite endpoint events including in-hospital and out-of-hospital death and/or readmission due to recurrent myocardial infarction (MI) or exacerbated symptoms of heart failure following MI.

**Results:** After 6-month follow-up, a total of 197 AMI patients were available and divided in two groups according to good prognosis (*n* = 144) and poor prognosis (*n* = 53). Our data identified serum myoglobin ≥651 ng/mL, serum creatinine ≥96 μM, Killip classification 2–4, and female gender as independent predictors of 6-month mortality and/or readmission after AMI. Moreover, we demonstrated that Killip classification 2–4 combined with either myoglobin (AUC_Killip class 2−4+myoglobin_ = 0.784, sensitivity = 69.8%, specificity = 79.9%) or creatinine (AUC_Killip class 2−4+creatinine_ = 0.805, sensitivity = 75.5%, specificity = 77.1%) could further enhance the predictive capacity of poor 6-month prognosis among AMI patients.

**Conclusions:** Patients with AMI ranked in the higher Killip class need to be evaluated and monitored with attention. Multibiomarker approach using Killip classification 2–4 and myoglobin or creatinine may be an effective way for 6-month prognosis prediction in AMI patients.

## Introduction

Acute myocardial infarction (AMI) is among the leading causes of death worldwide (1, 2). Despite that great progress has been made in the pharmacological and interventional therapy of AMI, patients with AMI may have the risk of developing recurrent cardiovascular events leading to rehospitalization or even death (3, 4). A deep understanding of the prediction factors of AMI prognosis can provide important information for disease stratification and clinical treatment of patients.

Because of the rapid advancement of laboratory techniques, a number of biomarkers have been identified for diagnosis of AMI. Among the biomarkers of cardiac necrosis injury, cardiac troponins, especially troponin I (cTnI) and troponin T, are considered as highly specific and sensitive markers of AMI diagnosis (5). Myoglobin and creatine kinase-MB (CK-MB), although with less specificity, are also valuable diagnostic biomarkers because of their rapid elevation in the early stage of AMI (6). For prognosis of AMI, cardiac troponins, brain natriuretic peptide (BNP), and N-terminal pro–brain natriuretic peptide (NT-proBNP) were proved to have prognostic values of heart failure and/or mortality in AMI patients (7, 8). In addition, increased heart-type fatty acid–binding protein and C-reactive protein (CRP) levels were reported to be possibly predictive of heart failure or mortality during the follow-up of AMI patients (9, 10). However, there is actually no gold standard prognostic biomarker for AMI (11). Multiple factors such as demographics, clinical presentations, and comorbidities are associated with AMI prognosis (12–14). Clinical studies are still highly needed to evaluate the factors predicting prognosis of AMI, especially with continuous advances in cardiovascular care (15).

In the present study, we aimed to analyze the prediction factors of poor prognosis (mortality and/or readmission) of AMI patients during a 6-month follow-up.

## Patients and Methods

### Patients

A total of 206 consecutive patients hospitalized for the first visit with AMI were enrolled from October 2015 to August 2017 at Department of Cardiology in Tongji Hospital affiliated to Tongji University (Shanghai, China). This cohort of patients was previously used to analyze gender-specific predictive markers of poor AMI prognosis, which was an independent analysis from the present study (16). The diagnosis of AMI was made by cardiologists according to Guidelines for the Diagnosis and Treatment of AMI in China. Those with malignant tumors, severe mental illness, and/or uncontrolled systemic diseases were excluded from the present study. The study protocol was approved by the independent ethics committee of Tongji Hospital affiliated to Tongji University (Shanghai, China). The written informed consent form was provided by all patients.

### Data Collection Flow

Data collection included demographic characteristics, medical history, clinical information, laboratory results, and oral medications within 24 h of admission. At admission or on the next morning, venous blood was taken and immediately analyzed in the Core Laboratory of Tongji Hospital for examinations of blood biochemistry, markers of myocardium injury (e.g., CK, CK-MB, myoglobin, cTnI, NT-proBNP), CRP, hemoglobin A_1c_ (HbA_1c_), glycated serum albumin (GSA), d-dimer, and folic acid.

### Follow-Up and Primary Endpoint

At 1, 3, and 6 months after discharge, AMI patients were followed up to assess the occurrence of composite endpoint events by trained researchers. The primary endpoint events were a composite of all-cause mortality (including in-hospital and out-of-hospital death) and/or readmission due to recurrent myocardial infarction (MI) or exacerbated symptoms of heart failure following MI. During 6-month follow-up, nine patients (4.4%) were lost over time as reported before, because they provided wrong telephone number or disconnected the call (16). The primary endpoint events were eventually confirmed by patients themselves, their families, and local hospital doctors. For analysis of predictive markers, AMI patients were divided into good prognosis vs. poor prognosis groups according to the occurrence of death and/or readmission.

### Statistical Analysis

Statistical analysis was performed using SPSS version 25.0 (SPSS Inc., Chicago, IL, USA) and MedCalc version 19.0.2 (MedCalc Software, Mariakerke, Belgium). Continuous variables with normal distribution were presented as mean ± standard deviation. For the cases of skewed distribution, median with interquartile range would be selected. All categorical variables and frequency of events were shown as numbers (percentage). The comparison between groups was performed with the independent-samples *t*-test, Mann-Whitney *U* test, or χ^2^ test as appropriate. Based on the significant (*P* < 0.05) variables between good prognosis and poor prognosis groups, forward stepwise COX regression analyses (entry only if *P* ≤ 0.10 and removal only if *P* > 0.10) were further applied to identify the independent predictors of 6-month prognosis. Receiver operating characteristic (ROC) curves and Kaplan–Meier curves were then constructed to determine the cutoff point and predictive value of these markers in the prediction of poor AMI prognosis. *P* < 0.05 was considered as statistically significant.

## Results

### Clinical Characteristics of AMI Patients

After 6-month follow-up, a total of 197 AMI patients were available and divided into two groups according to prognosis. Those with death and/or readmission events (*n* = 53) were defined as poor prognosis, whereas the other AMI patients without death and/or readmission (*n* = 144) were defined as good prognosis (Table 1). In patients with poor prognosis (*n* = 53), 41 patients were rehospitalized (including three patients died during readmission), and 15 patients died (including both in-hospital and out-of-hospital death). Demographics showed that AMI patients with poor prognosis were older than those with good prognosis (71.3 ± 14.1 vs. 61.7 ± 13.3 years, *P* < 0.001). Patients with AMI with poor prognosis also had lower diastolic blood pressure (70.5 ± 13.5 mmHg vs. 75.2 ± 12.2 mmHg, *P* < 0.05), less current or past smoking experience (43.4 vs. 61.8%, *P* < 0.05), and higher prevalence of diabetes mellitus (41.5 vs. 21.5%, *P* < 0.01) when compared to those with good prognosis. A significantly larger proportion of AMI patients with poor prognosis had Killip classification 2–4 compared to those with good prognosis (69.8 vs. 20.1%, *P* < 0.001). Meanwhile, AMI prognosis was analyzed according to different genders, which showed that the proportion of AMI patients with poor prognosis (including death and/or readmission) was 45.0% in females (*n* = 18 among 40 females), which was significantly higher than 22.3% in males (*n* = 35 among 157 males) (*P* < 0.01).

**Table 1:** Baseline clinical characteristics of patients.

	**All patients** ** (*n* = 197)**	**Good prognosis** ** (*n* = 144)**	**Poor prognosis** ** (*n* = 53)**	***P*-value**
**Demographic characteristics**			
Age, years	64.3 ± 14.2	61.7 ± 13.3	71.3 ± 14.1	<0.001
BMI, kg/m^2^	24.8 ± 3.2	24.8 ± 3.2	24.7 ± 3.5	0.715
SBP, mmHg	126.0 ± 24.2	127.7 ± 23.9	121.5 ± 24.4	0.109
DBP, mmHg	73.9 ± 12.7	75.2 ± 12.2	70.5 ± 13.5	0.022
Heart rate, bpm	79.5 ± 16.0	78.5 ± 14.9	82.1 ± 18.6	0.162
Current or past smoker, *n* (%)	112 (56.9%)	89 (61.8%)	23 (43.4%)	0.021
**Gender**, ***n*** **(%)**			
Male	157 (79.7)	122 (84.7)	35 (66.0)	0.004
Female	40 (20.3)	22 (15.3)	18 (34.0)	
**Classification of AMI**, ***n*** **(%)**			
STEMI	167 (84.8)	120 (83.3)	47 (88.7)	0.354
NSTEMI	30 (15.2)	24 (16.7)	6 (11.3)	
**Previous history**, ***n*** **(%)**			
Hypertension	127 (64.5)	91 (63.2)	36 (67.9)	0.538
Diabetes mellitus	53 (26.9)	31 (21.5)	22 (41.5)	0.005
Atrial fibrillation	12 (6.1)	7 (4.9)	5 (9.4)	0.393
Stroke	35 (17.8)	25 (17.4)	10 (18.9)	0.806
**Killip classification**, ***n*** **(%)**			
Killip classification 2–4	66 (33.5)	29 (20.1)	37 (69.8)	<0.001

*BMI, body mass index; SBP, systolic blood pressure; DBP, diastolic blood pressure; AMI, acute myocardial infarction; STEMI, ST-segment elevation myocardial infarction; NSTEMI, non–ST-segment elevation myocardial infarction*.

Oral medications were recorded at admission. Among the commonly used medicine, a larger proportion of AMI patients in poor prognosis group were prescribed with loop diuretics compared to those with good prognosis (41.5 vs. 12.5%, *P* < 0.001). No difference was found in other oral medications, including angiotensin-converting enzyme inhibitor/angiotensin receptor blocker, β-blockers, antiplatelet drugs, anticoagulant drugs, and statins, between the good prognosis and poor prognosis groups (Table 2).

**Table 2:** Oral medications at admission.

	**All patients** ** (*n* = 197)**	**Good prognosis** ** (*n* = 144)**	**Poor prognosis** ** (*n* = 53)**	***P*-value**
Loop diuretics, *n* (%)	40 (20.3)	18 (12.5)	22 (41.5)	<0.001
ACEI/ARB, *n* (%)	128 (65.0)	99 (68.8)	29 (55.8)	0.092
β-Blockers, *n* (%)	128 (65.0)	99 (68.8)	29 (55.8)	0.092
Antiplatelet drugs, *n* (%)	196 (99.5)	144 (100.0)	52 (98.1)	1.000
Anticoagulant drugs, *n* (%)	87(44.2)	68 (47.2)	19 (36.5)	0.184
Statins, *n* (%)	192 (97.5)	142 (98.6)	50 (94.3)	0.859

*ACEI, angiotensin-converting enzyme inhibitor; ARB, angiotensin receptor blocker*.

### Biochemical Examinations of AMI Patients

At admission or on the next morning, biochemical examinations were performed for AMI patients and were further compared between AMI patients with good and poor prognosis (Table 3). Compared to those with good prognosis, patients with poor prognosis were present with slightly lower serum levels of albumin, hemoglobin, and sodium, but higher levels of CRP, blood urea nitrogen (BUN), creatinine, and uric acid. Most of the biochemical data mentioned above had a mean value or median value within the normal range, except for BUN, creatinine, and uric acid, which were slightly above the normal range in the group of AMI patients with poor prognosis. Meanwhile, AMI patients had obviously higher levels of CK, CK-MB, myoglobin, cTnI, and NT-proBNP. Among these biochemical data indicating myocardial necrosis and heart failure, myoglobin and NT-proBNP were significantly elevated in AMI patients with poor prognosis. In particular, AMI patients with poor prognosis had a median value of NT-proBNP more than 5-fold higher than those with good prognosis [1,517.3–12,885.3 (4,448.5) U/L vs. 388.4–1,619.8 (788.4) U/L, *P* < 0.001]. Moreover, AMI patients with poor prognosis had slightly higher levels of HbA_1c_, GSA, and d-dimer when compared to those with good prognosis.

**Table 3:** Biochemical examinations of patients.

	**All patients** ** (*n* = 197)**	**Good prognosis** ** (*n* = 144)**	**Poor prognosis** ** (*n* = 53)**	***P*-value**
Albumin, g/dL	3.7 ± 0.4	3.8 ± 0.4	3.6 ± 0.5	0.015
Hemoglobin, g/dL	13.2 ± 1.9	13.5 ± 1.6	12.3 ± 2.2	<0.001
Sodium, mM	137.7–140.9 (139.4)	137.9–141.1 (139.5)	136.7–140.6 (138.4)	0.027
Potassium, mM	3.9 ± 0.5	3.8 ± 0.4	4.0 ± 0.7	0.062
BUN, mM	4.5–7.0 (5.5)	4.2–6.3 (5.3)	5.2–11.6 (7.8)	<0.001
LDL-C, mM	3.3 ± 0.8	3.3 ± 0.8	3.1 ± 0.9	0.151
HDL-C, mM	1.03 ± 0.21	1.03 ± 0.21	1.00 ± 0.22	0.311
CRP, mg/dL	0.3–2.0 (0.8)	0.3–1.4 (0.7)	0.5–5.1 (1.4)	<0.001
CK, U/L	543.5–2,574.0 (1,162.0)	559.5–2,685.3 (1,179.5)	505.0–2,134.0 (1,048.0)	0.649
Myoglobin, ng/mL	141.5–644.9 (317.9)	130.4–567.4 (286.0)	201.0–935.8 (470.0)	0.004
CTnI, ng/mL	20.9–78.0 (63.9)	17.7–78.0 (54.1)	27.2–78.0 (75.0)	0.377
CK-MB, ng/mL	72.8–299.0 (194.2)	73.6–300.0 (193.3)	61.6–295.0 (197.1)	0.367
HbA_1c_, %	5.7–6.6 (6.0)	5.6–6.4 (5.9)	5.8–7.0 (6.1)	0.008
GSA, %	12.6–16.1 (14.3)	12.5–15.5 (13.9)	13.3–17.9 (15.3)	0.002
Creatinine, μM	77.0–101.0 (88.0)	74.0–95.0 (85.5)	83.0–140.0 (101.0)	<0.001
NT-proBNP, U/L	437.4–2,653.8 (1,037.5)	388.4–1,619.8 (788.4)	1,517.3–12,885.3 (4,448.5)	<0.001
Uric acid, μM	393.8 ± 121.6	372.4 ± 101.0	451.4 ± 151.6	0.001
ALT, U/L	28.0–68.5 (39.0)	28.0–66.5 (38.0)	29.5–78.5 (43.0)	0.407
AST, U/L	79.0–296.5 (158.0)	75.0–297.3 (157.5)	80.5–286.5 (165.0)	0.964
d-Dimer, mg/L	0.3–0.9 (0.4)	0.2–0.7 (0.4)	0.4–2.4 (0.7)	<0.001
Folic acid, mM	7.5 ± 3.7	7.7 ± 3.7	7.1 ± 3.8	0.365

*BUN, blood urea nitrogen; LDL-C, low-density lipoprotein cholesterol; HDL-C, high-density lipoprotein cholesterol; CRP, C-reactive protein; CK, creatine kinase; CK-MB, creatine kinase MB; cTnI, cardiac troponin I; HbA_1c_, hemoglobin A1c; GSA, glycated serum albumin; NT-proBNP, N-terminal pro–brain natriuretic peptide; ALT, alanine aminotransferase; AST, aspartate transaminase*.

### Myoglobin, Creatinine, Killip Classification 2–4, and Gender Are Independent Predictors of Poor AMI Prognosis

We then constructed multivariate COX stepwise regression analysis to identify the independent predictors of poor AMI prognosis during 6-month follow-up in the present study. With stepwise variable selection using the covariates based on the significant (*P* < 0.05) variables in baseline characteristics and biochemical data between good vs. poor prognosis groups, myoglobin, creatinine, Killip classification 2–4, and gender were identified as potential independent predictors of poor AMI prognosis (Table 4).

**Table 4:** Forward stepwise COX regression analysis (entry only if *P* ≤ 0.10 and removal only if *P* > 0.10) for poor AMI prognosis.

	**β Value**	**Standard error**	**Hazards ratio**	**95% CI**	***P*-value**
**Variables in the Equation (Entry Only if** ***P*** **≤** **0.10)**					
Myoglobin	0.000	0.000	1.000	1.000–1.001	0.005
Creatinine	0.011	0.003	1.011	1.006–1.016	<0.001
CRP	0.007	0.004	1.007	0.999–1.014	0.098
Killip class 2–4	1.420	0.377	4.139	1.976–8.672	<0.001
Gender	−0.934	0.354	0.393	0.196–0.787	0.008
	**Score**		**Degrees of freedom**		***P*****-value**
**Variables not in the Equation (Removal Only if** ***P*** **>** **0.10)**					
Age	2.068		1		0.150
DBP	2.393		1		0.122
Current or past smoker	0.230		1		0.631
Diabetes mellitus	0.567		1		0.451
Loop diuretics	0.480		1		0.488
Albumin	0.081		1		0.776
Hemoglobin	0.001		1		0.971
Sodium	0.379		1		0.538
BUN	0.441		1		0.506
NT-proBNP	1.156		1		0.282
HbA_1c_	0.537		1		0.464
GSA	0.694		1		0.405
Uric acid	0.082		1		0.775
d-Dimer	0.340		1		0.560

*CRP, C-reactive protein*.

*DBP, diastolic blood pressure; BUN, blood urea nitrogen; NT-proBNP, N-terminal pro–brain natriuretic peptide; HbA_1c_, hemoglobin A_1c_; GSA, glycated serum albumin*.

**Figure 1:**
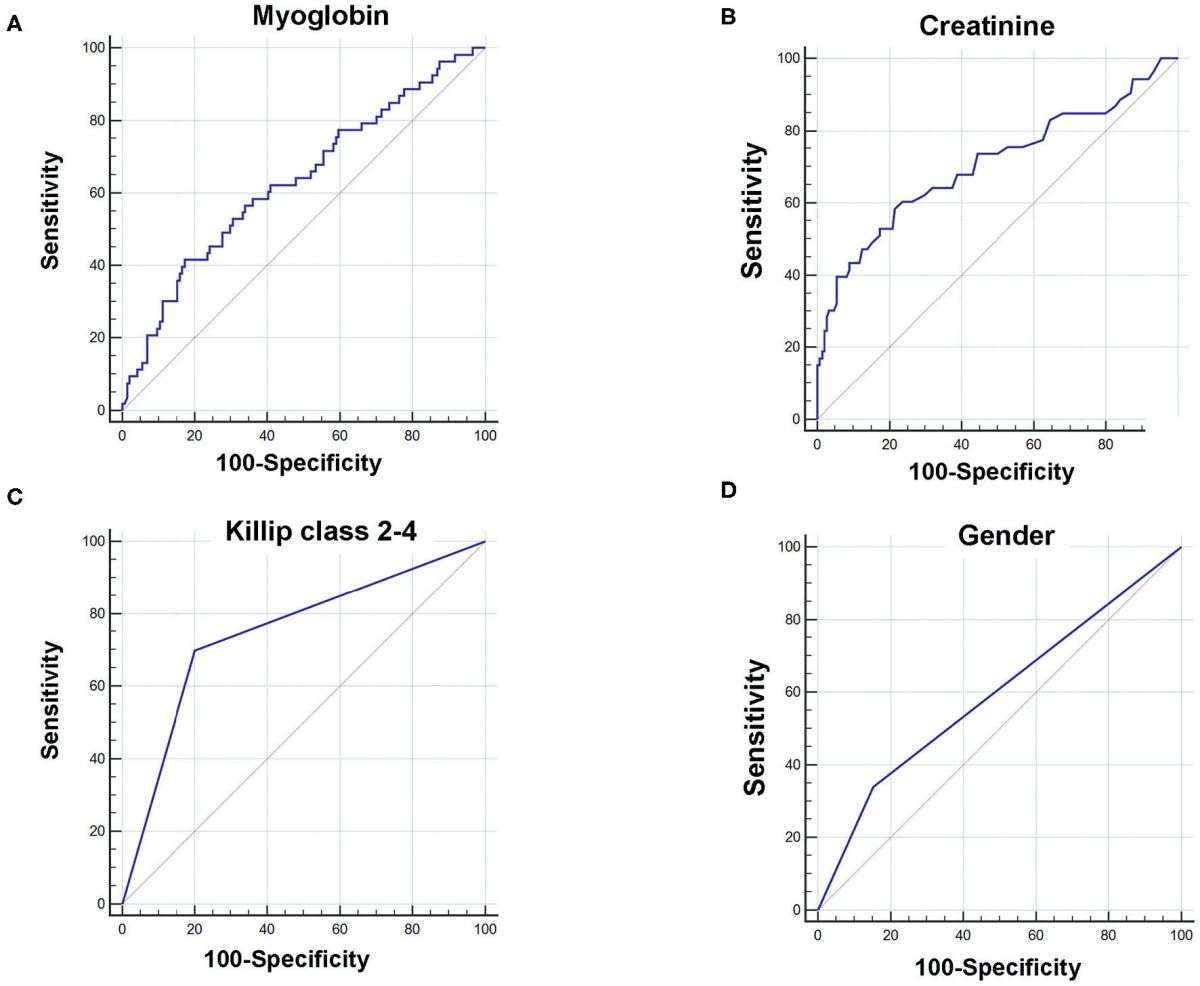
Receiver operating characteristic curve (ROC) of myoglobin, creatinine, Killip classification 2–4, and gender for predicting 6-month prognosis in AMI patients. **(A)** AUC_myoglobin_ = 0.632, 95% CI = 0.561–0.700, sensitivity = 41.5%, specificity = 82.6%, cutoff point: 651; **(B)** AUC_creatinine_ = 0.706, 95% CI = 0.637–0.768, sensitivity = 58.5%, specificity = 78.5%, cutoff point: 96; **(C)** AUC_Killip class 2−4_ = 0.748, 95% CI = 0.682–0.807, sensitivity = 69.8%, specificity = 79.9%. **(D)** AUC_gender_ = 0.593, 95% CI = 0.521–0.663, sensitivity = 34.0%, specificity = 84.7%.

**Figure 2:**
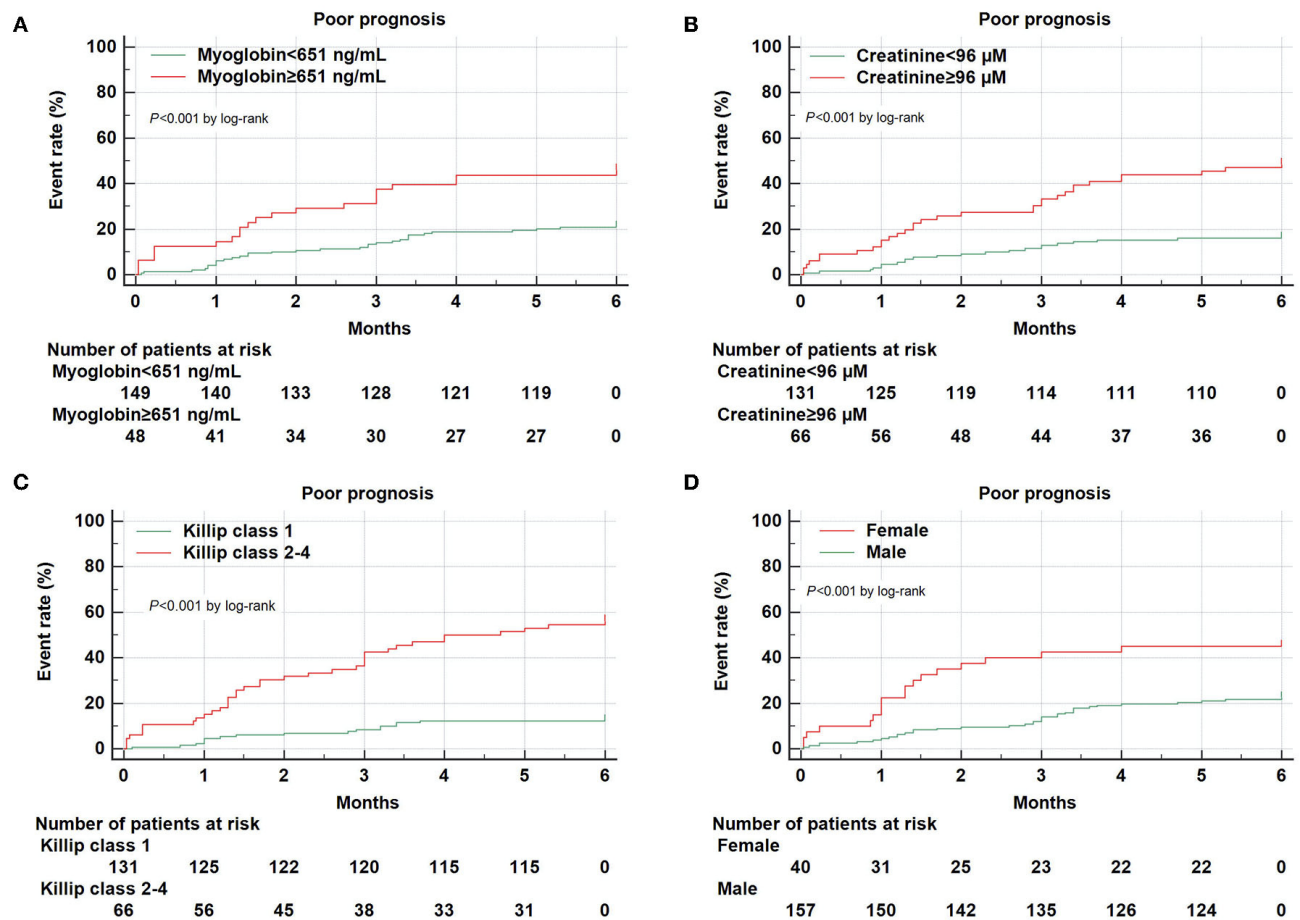
Kaplan–Meier survival curves for 6-month prognosis of AMI patients. Kaplan–Meier survival curves were constructed according to myoglobin **(A)**, creatinine **(B)**, Killip classification 2–4 **(C)**, and gender **(D)**.

Receiver operating characteristic curves further demonstrated that area under the curve (AUC) was AUC_myoglobin_ = 0.632 (95% CI = 0.561–0.700, sensitivity = 41.5%, specificity = 82.6%), AUC_creatinine_ = 0.706 (95% CI = 0.637–0.768, sensitivity = 58.5%, specificity = 78.5%), AUC_Killip class 2−4_ = 0.748 (95% CI = 0.682–0.807, sensitivity = 69.8%, specificity = 79.9%), and AUC_gender_ = 0.593 (95% CI = 0.521–0.663, sensitivity = 34.0%, specificity = 84.7%), respectively (Figure 1). Using the cutoff point calculated from ROC analysis, myoglobin ≥651 ng/mL, creatinine ≥96 μM, Killip classification 2–4, and female gender were found to be significant predictors of poor prognosis in AMI patients (Figure 2).

### Combined Analysis of Independent Predictors of Poor AMI Prognosis

**Figure 3:**
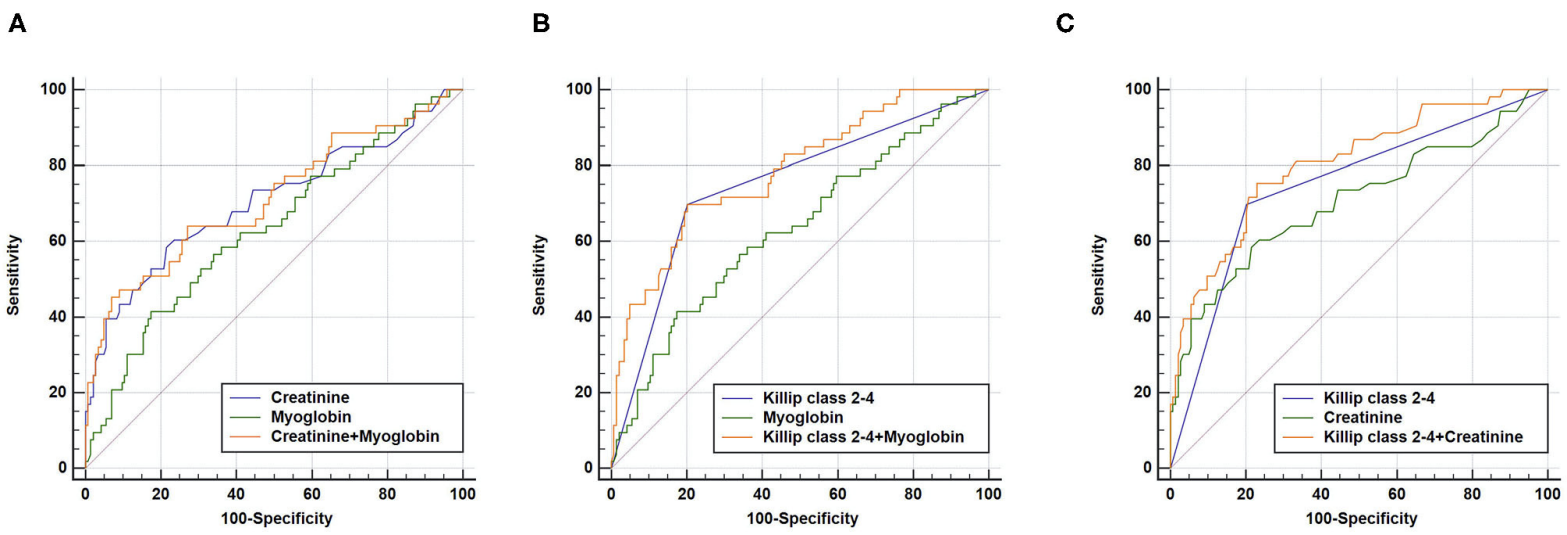
Combination of two independent predictors in construction of Receiver operating characteristic (ROC) curve. **(A)** AUC_creatinine+myoglobin_ = 0.713, 95% CI = 0.645–0.775, sensitivity = 45.3%, specificity = 93.1%, AUC_creatinine+myoglobin_ vs. AUC_creatinine_ (*P* = 0.5736), AUC_creatinine+myoglobin_ vs. AUC_myoglobin_ (*P* = 0.1094); **(B)** AUC_Killip class 2−4+myoglobin_ = 0.784, 95% CI = 0.720–0.839, sensitivity = 69.8%, specificity = 79.9%, AUC_Killip class 2−4+myoglobin_ vs. AUC_Killip class 2−4_ (*P* = 0.0369), AUC_Killip class 2−4+myoglobin_ vs. AUC_myoglobin_ (*P* = 0.0003); **(C)** AUC_Killip class 2−4+creatinine_ = 0.805, 95% CI = 0.743–0.858, sensitivity = 75.5%, specificity = 77.1%, AUC_Killip class 2−4+creatinine_ vs. AUC_Killip class 2−4_ (*P* = 0.0108), AUC_Killip class 2−4+creatinine_ vs. AUC_creatinine_ (*P* = 0.0038).

We further performed combined analysis of these independent markers to explore whether this could further enhance their predictive capacity for poor AMI prognosis (Figure 3). We found that a combination of Killip classification 2–4 with myoglobin was sufficient to enhance AUC (AUC_Killip class 2−4+myoglobin_ = 0.784, 95% CI = 0.720–0.839, sensitivity = 69.8%, specificity = 79.9%) when compared to AUC_Killip class 2−4_ or AUC_Myoglobin_ alone (*P* < 0.05 and *P* < 0.001, respectively). Similarly, a combination of Killip classification 2–4 with creatinine was also able to enhance AUC (AUC_Killip class 2−4+creatinine_ = 0.805, 95% CI = 0.743–0.858, sensitivity = 75.5%, specificity = 77.1%) when compared to AUC_Killip class 2−4_ or AUC_creatinine_ alone (*P* < 0.05 and *P* < 0.01, respectively).

**Figure 4:**
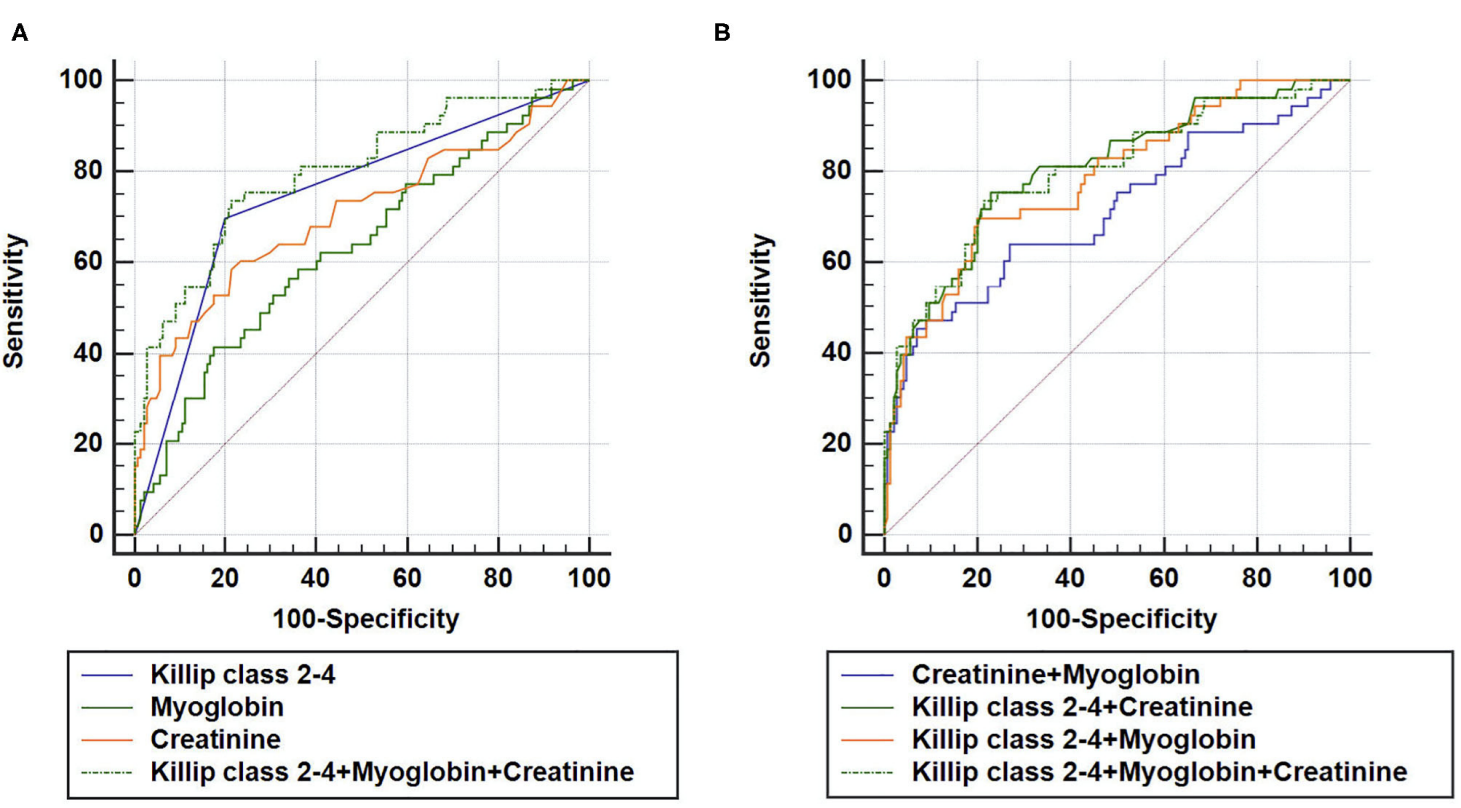
Combination of three independent predictors in construction of Receiver operating characteristic (ROC) curve. AUC_Killip class 2−4+creatinine+myoglobin_ = 0.800, 95% CI = 0.737–0.853, sensitivity = 73.6%, specificity = 78.5%. **(A)** AUC_Killip class 2−4+myoglobin+creatinine_ vs. AUC_Killip class 2−4_ (*P* = 0.0224), AUC_Killip class 2−4+myoglobin+creatinine_ vs. AUC_myoglobin_ (*P* = 0.0007), AUC_Killip class 2−4+myoglobin+creatinine_ vs. AUC_creatinine_ (*P* = 0.0088). **(B)** AUC_Killip class 2−4+myoglobin+creatinine_ vs. AUC_creatinine+myoglobin_ (*P* = 0.0056), AUC_Killip class 2−4+myoglobin+creatinine_ vs. AUC_Killip class 2−4+creatinine_ (*P* = 0.3557), AUC_Killip class 2−4+myoglobin+creatinine_ vs. AUC_Killip class 2−4+myoglobin_ (*P* = 0.4398).

As expected, a combination of Killip class 2–4, myoglobin, and creatinine could also increase AUC (AUC_Killip class 2−4+myoglobin+creatinine_ = 0.800, 95% CI = 0.737–0.853, sensitivity = 73.6%, specificity = 78.5%) compared to either predictive marker alone (Figure 4). Noteworthy, although AUC_Killip class 2−4+myoglobin+creatinine_ was significantly larger than AUC_creatinine+myoglobin_, no significant difference was found for AUC_Killip class 2−4+myoglobin+creatinine_ when compared to AUC_Killip class 2−4+myoglobin_ or AUC_Killip class 2−4+creatinine_. Taken together, these data suggest that a combination of Killip classification 2–4 either with myoglobin (AUC_Killip class 2−4+myoglobin_) or creatinine (AUC_Killip class 2−4+creatinine_) was sufficient to enhance the predictive capacity for AMI poor prognosis.

## Discussion

A great number of AMI patients are at risk of recurrent cardiovascular events, which leads to readmission or even dearth. Biomarkers are useful in the prediction of AMI prognosis, which may differ from endpoint events and follow-up durations. Based on a cohort of 197 AMI patients followed up for 6 months, our study shows that serum myoglobin ≥651 ng/mL, serum creatinine ≥96 μM, Killip classification 2–4, and female gender are independent predictors of 6-month mortality and/or readmission. Our data also demonstrate that the combination of Killip classification 2–4 either with creatinine or myoglobin could further enhance the predictive capacity for AMI poor prognosis.

Demographic characteristics and oral medications at admission were first compared in AMI patients with good prognosis (*n* = 144) and poor prognosis (*n* = 53). Compared to those with good prognosis, patients with poor prognosis were about 10 years older and more likely to have previous history of diabetes mellitus. Aging and diabetes are both well-known risk factors for worse outcomes after MI that have strong associations with death or recurrent cardiovascular events (17, 18). The increased risk of adverse outcomes in patients with older age or diabetes is likely multifactorial, which may be explained by more complicating diseases, higher prevalence of multivessel disease, and less implementation of evidence-based therapies (19–21).

Gender-related difference exists in the assessment, treatment, and outcomes of coronary artery diseases (22, 23). Based on the same cohort of AMI patients followed up for 6 months, we previously analyzed and reported gender-specific predictive markers of poor AMI prognosis in male and female patients; in that study, although there was no significant difference in 6-month mortality between males and females, the readmission rate was significantly higher in females than in males (16). Here, when we analyzed death and readmission events together in AMI patients, we observed that 45.0% of females had death and/or readmission events during 6-month follow-up, which was significantly higher than 22.3% in males. COX regression analyses and Kaplan–Meier curves further demonstrated that female gender was an independent predictor of 6-month mortality and/or death after AMI. The predictive value of female gender in AMI prognosis may differ by age as well as by endpoint events analyzed in different studies. A great number of studies support that younger female patients with AMI were at higher risk of both short- and long-term mortality than male patients; however, this difference was diminished in the old population (24–26). Additionally, most studies used mortality as endpoint event (27–29), whereas our study defined poor prognosis including both death and hospital readmission. Indeed, a deeper understanding of the impact of gender on the outcomes of AMI may help guide better therapeutic strategies for male and female patients.

Biochemical examination data were then analyzed between AMI patients who had good vs. poor prognosis. Among the biochemical parameters different between good and poor prognosis groups, serum myoglobin and serum creatinine were found to be independent indicators for death and/or readmission among AMI patients. Myoglobin is a widely used biomarker for early diagnosis of MI that rises earlier than troponins. However, the diagnostic value of myoglobin is limited because of its less specificity to cardiomyocyte death. Indeed, a combined analysis of myoglobin, CK-MB, and cTnI is the most often used biochemical examination for MI diagnosis among suspected patients (30). In the present study, our data showed that serum myoglobin, CK-MB, and cTnI were all markedly elevated in AMI patients. However, only myoglobin was proved to be an independent predictor for poor AMI prognosis. It was previously reported that elevated myoglobin was a predictive biomarker better than cTnI for 5-year mortality in patients evaluated in the emergency department for possible acute coronary syndromes (ACS) (31). In comparison to the long-term prognosis of patients with undifferentiated chest pain, our study analyzed the short-term prognosis of AMI patients and observed that myoglobin ≥651 ng/mL was predictive for 6-month poor prognosis after AMI. Our findings were consistent with previous studies that reported the prognostic value of myoglobin to predict mortality in patients with ACS (32) and MI (33). Indeed, despite the absence of cardiac specificity, the prognostic value of myoglobin and its cutoff point for clinical use of predicting mortality and/or readmission after AMI deserve further investigation.

Additionally, we observed that serum creatinine level was significantly higher in AMI patients with poor prognosis [83.0–140.0 (101.0) μM] than those with good prognosis [74.0–95.0 (85.5) μM]. Furthermore, serum creatinine ≥96 μM was able to independently predict 6-month death and/or readmission among AMI patients. Serum creatinine is a commonly used biomarker for kidney function. In addition to its ability to reflect kidney dysfunction, serum creatinine has been demonstrated to be an independent predictor for in-hospital and out-of-hospital mortality among patients with ACS or AMI (34, 35). In addition, subclinical serum creatinine elevation was reported to have prognostic value of adverse in-hospital outcomes among MI patients, which was independent of baseline renal function (36). The predictive value of high creatinine level for poor AMI prognosis is usually closely related to the kidney damage or dysfunction developed in those patients with impaired cardiac function (37). In our study, the median value of serum creatinine was slightly above the normal range in the group of AMI patients with poor prognosis compared to those with good prognosis. A cutoff point ≥96 μM was found to independently predict 6-month death and/or readmission among AMI patients. These data suggest that the serum creatinine elevation (even moderate elevation) also needs to be taken seriously in clinical evaluation, which may provide important information for poor prognosis among AMI patients.

Killip classification is usually evaluated for AMI patients, which is classified as Killip 1 (without heart failure), Killip 2 (with mild heart failure), Killip 3 (with pulmonary edema), and Killip 4 (with cardiogenic shock). Increasing evidence has indicated that higher Killip classification is associated with poor prognosis in patients with ACS (38, 39). Killip class ≥2 at presentation was previously found to be an independent predictor of in-hospital and long-term mortality of AMI patients (40, 41). Additionally, AMI patients were reported to have higher glucose level at presentation (42). In the present study, Killip classification 2–4 was identified as a strong independent predictor of 6-month death and/or readmission among AMI patients (AUC_Killip class 2−4_ = 0.748, 95% CI = 0.682–0.807, sensitivity = 69.8%, specificity = 79.9%). Moreover, our data showed that Killip classification 2–4 combined with either myoglobin (AUC_Killip class 2−4+myoglobin_ = 0.784, 95% CI = 0.720–0.839, sensitivity = 69.8%, specificity = 79.9%) or creatinine (AUC_Killip class 2−4+creatinine_ = 0.805, 95% CI = 0.743–0.858, sensitivity = 75.5%, specificity = 77.1%) further enhanced the predictive capacity for AMI poor prognosis. Our data, together with previous reports, highly suggest that it is important to better evaluate AMI patients ranked in the higher Killip classes. Multibiomarker approach could provide more information for the risk stratification of AMI. Moreover, AMI patients with Killip classification 2–4 need to be monitored and managed differently to improve the prognosis after AMI (43).

There are some limitations that need to be noted. First, the cohort of patients is not large enough. A relatively small number of female AMI patients were enrolled in the present study. Second, despite that the medication information was not available for each patient after 6-month follow-up, whether the medication was changed or not is important information that may also influence AMI prognosis (44). Third, the prognostic value of the four identified predictors and its cutoff point for clinical use of poor AMI prognosis deserve further investigation.

In conclusion, our study identifies serum myoglobin ≥651 ng/mL, serum creatinine ≥96 μM, Killip classification 2–4, and female gender as independent predictors of 6-month mortality and/or readmission after AMI. Noteworthy, Killip classification 2–4 combined with either myoglobin or creatinine further enhances the predictive capacity of poor AMI prognosis. Multibiomarker approach using Killip classification 2–4 and myoglobin or creatinine may be an effective way for 6-month prognosis prediction in AMI patients.

## Data Availability Statement

All datasets presented in this study are included in the article/supplementary material.

## Ethics Statement

The studies involving human participants were reviewed and approved by Ethics Committee of Tongji Hospital affiliated to Tongji University (Shanghai, China). The patients/participants provided their written informed consent to participate in this study.

## Author Contributions

JY collected clinical and biochemical data, took charge of follow-up, and analyzed the data. YX obtained informed consent from patients and participated in the follow-up of patients. YL and YT collected patient serum and ensured the quality of serum collection. JX designed and supervised the study and wrote the paper. All authors contributed to the article and approved the submitted version.

## Conflict of Interest

The authors declare that the research was conducted in the absence of any commercial or financial relationships that could be construed as a potential conflict of interest.
